# Circulating cytokines and procalcitonin in acute Q fever granulomatous hepatitis with poor response to antibiotic and short-course steroid therapy: a case report

**DOI:** 10.1186/1471-2334-10-193

**Published:** 2010-07-01

**Authors:** Chung-Hsu Lai, Jiun-Nong Lin, Lin-Li Chang, Yen-Hsu Chen, Hsi-Hsun Lin

**Affiliations:** 1 Division of Infectious Diseases, Department of Internal Medicine, E-Da Hospital/I-Shou University, Kaohsiung County, Taiwan; 2 Graduate Institute of Medicine, College of Medicine, Kaohsiung Medical University, Kaohsiung City, Taiwan; 3 Faculty of Medicine, Department of Microbiology, College of Medicine, Kaohsiung Medical University, Kaohsiung City, Taiwan; 4 Division of Infectious Diseases, Department of Internal Medicine, Kaohsiung Medical University Hospital, Kaohsiung Medical University, Kaohsiung City, Taiwan; 5 Graduate Institute of Medicine, Tropical Medicine Research Institute, College of Medicine, Kaohsiung Medical University, Kaohsiung City, Taiwan; 6 Institute of Clinical Medicine, National Yang-Ming University, Taipei City, Taiwan

## Abstract

**Background:**

Q fever is a zoonosis distributed worldwide that is caused by *Coxiella burnetii *infection and the defervescence usually occurs within few days of appropriate antibiotic therapy. Whether the changes of cytokine levels are associated with acute Q fever with persistent fever despite antibiotic therapy had not been investigated before.

**Case Presentation:**

We report a rare case of acute Q fever granulomatous hepatitis remained pyrexia despite several antibiotic therapy and 6-day course of oral prednisolone. During the 18-month follow-up, the investigation of the serum cytokines profile and procalcitonin (PCT) revealed that initially elevated levels of interleukin-2 (IL-2), IL-8, IL-10, and PCT decreased gradually, but the IL-6 remained in low titer. No evidence of chronic Q fever was identified by examinations of serum antibodies against *C. burnetii *and echocardiography.

**Conclusions:**

The changes of cytokine levels may be associated with acute Q fever with poor response to treatment and PCT may be an indicator for monitoring the response to treatment.

## Background

Q fever is a zoonosis distributed worldwide that is caused by *Coxiella burnetii *infection [1]. The major clinical manifestations of symptomatic acute Q fever are influenza-like illness with various degrees of pneumonia or hepatitis [1]. Defervescence usually occurs several days after the administration of antibiotics that are effective against *C. burnetii*. However, cases of acute Q fever with poor response to antimicrobial therapy that demand a combination therapy with steroids have been reported [2-4]. Development of autoantibodies that induce inflammatory or immunologic processes is presumed to be responsible for persistent fever [1-4]. Whether the changes of cytokine levels are associated with acute Q fever with persistent fever despite antibiotic therapy had not been investigated before. Herein, we report a case of acute Q fever granulomatous hepatitis with poor response to antibiotic and short-course steroid therapy, and we present the changes in the profile of circulating cytokines and procalcitonin (PCT), which was identified in a study conducted to investigate cytokine profiles in patients with Q fever.

## Case Presentation

A 35-year-old male was admitted because of a 7-day history of fever with chills. He was a truck driver and worked in southern Taiwan. He recalled a history of visiting his father, who bred hundreds of goats, about 3 weeks prior admission, but the patient denied direct contact with or close proximity to the goats.

Upon admission, the patient's body temperature was 39.7°C, his heart rate was 88 beats/min, and his blood pressure was 140/70 mmHg. No abnormality was revealed by physical examination. The laboratory examinations revealed a white blood cell (WBC) count of 10,450/mm^3 ^(neutrophils, 76%; lymphocytes, 16%; monocytes, 5%), a hemoglobin level of 12.7 g/dl, a platelet count of 324,000/mm^3^, an alanine aminotransferase (ALT) level of 145 U/L (reference range, 0-44 U/L), an aspartate aminotransferase (AST) level of 76 U/L (reference range, 0-38 U/L), a total bilirubin level of 0.9 mg/dl (reference, 0-1.3 mg/dl), and a serum creatinine level of 1.1 mg/dl. No abnormality was found by chest x-ray or urine analysis. Abdominal ultrasonography revealed hepatosplenomegaly, a moderately fatty liver, and a thickened gallbladder wall. Acute Q fever was highly suspected clinically, and oral doxycycline, 100 mg every 12 hours, was administered empirically. However, the fever persisted despite 4 days of treatment of doxycycline; thus, intravenous levofloxacin, 500 mg per day, was added starting at day 5. Abdominal computed tomography (CT) revealed multiple periaortic reactive lymph nodes, mesentery infiltration, and hepatomegaly. Neither mediastinal lymph nodes nor pulmonary lesion was found by chest CT. No focus of inflammation was found by Gallium inflammation scan. Oral prednisolone at a dosing schedule of 40 mg daily, 20 mg daily, and then 10 mg daily for 2 days each was administered from days 7 to 12. The first blood specimen collected on day 1 was sent to the Taiwan CDC for Q fever testing, and the results of the antibody assay and the polymerase chain reaction for *C. burnetii *were all reported to be negative on day 8.

**Figure 1:**
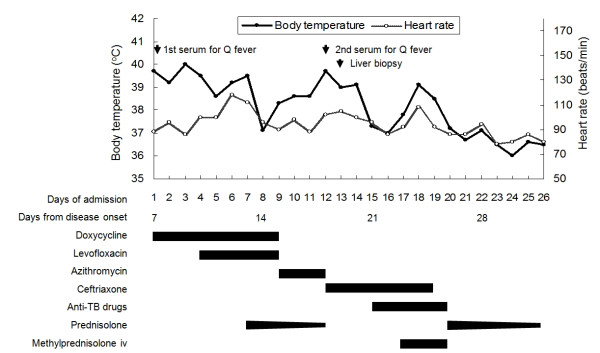
**Body temperature, heart rate, examinations, and treatment during the whole course of hospitalization**. The black bars indicate the duration of antibiotic and steroid therapy.

**Figure 2:**
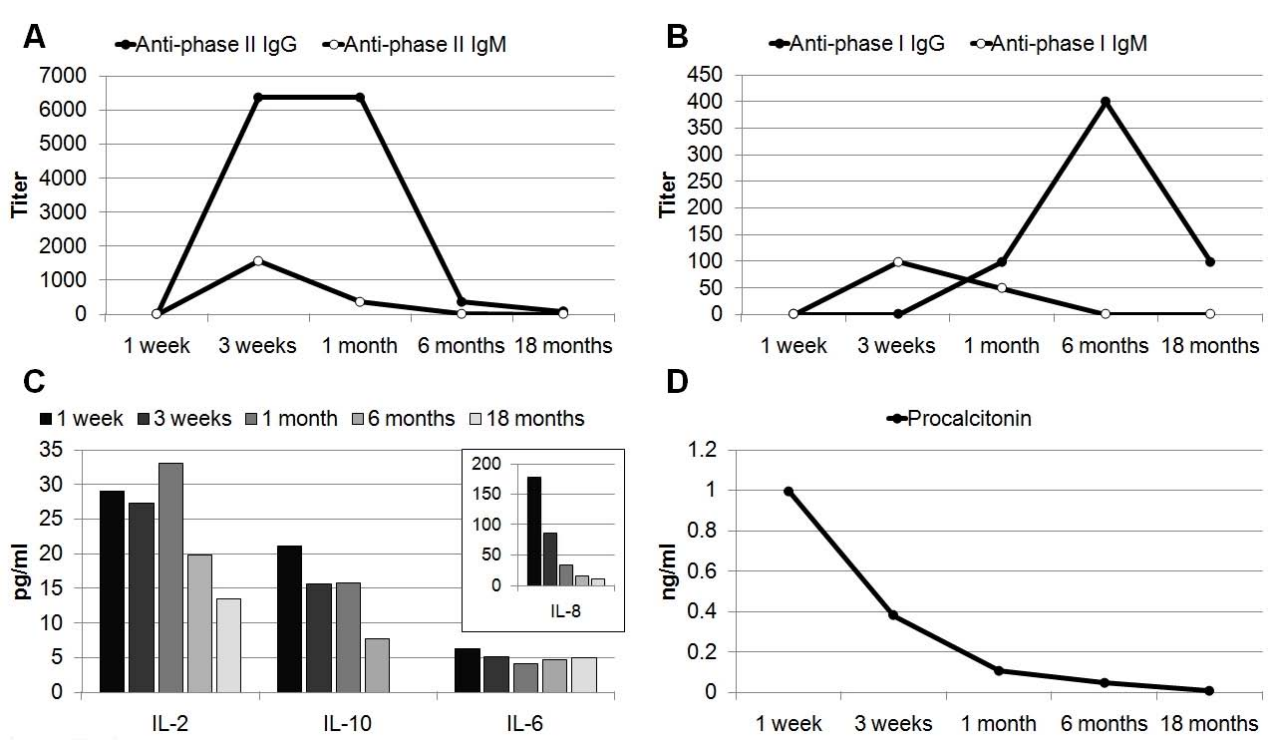
**Serial results of serum *C. burnetii *antibodies (A and B), cytokines (C), and procalcitonin (D) from disease onset to 18-months follow-up**.

No bacterial growth was found by blood and urine cultures. The results of the serum HBsAg, anti-HBc IgM, anti-HAV IgM, anti-HCV, anti-nuclear antibody, IgM antibodies against Epstein-Barr virus and Cytomegalovirus, anti-HIV, and VDRL tests were all negative. The thyroid function, serum cortisol level, and tumor markers including PSA, AFP, CEA, and Ca-199 were all within normal limits. Other antibiotics including azithromycin and ceftriaxones were administered later due to persistent fever. Because of the refractory spiking fever, hepatomegaly, elevated liver enzymes, and no identified infection source or pathogen, a sono-guided fine-needle liver biopsy was performed on day-13 (Figure 1). The pathological finding revealed granulomatous inflammation of the liver without the characteristic "doughnut granulomas" of Q fever. Neither acid-fast microorganisms nor fungus was found. No mycobacterium, fungus, or other bacterium was isolated from the tissue culture. Because tuberculosis (TB) is an endemic disease in southern Taiwan and is a common cause of granulomatous hepatitis, a therapeutic trial of four combined anti-TB drugs was added starting at day 15. Steroid therapy with intravenous methylprednisolone, 20 mg every 12 hours, was added 2 days later. On day 19, however, the Q fever analysis of serum collected on day 12 (19^th ^day from disease onset) revealed anti-phase II IgM = 1:1600 and anti-phase II IgG = 1:6400 (Figure 2A). At this time, the anti-TB drugs were suspended. The fever subsided, and the steroid was shifted to oral prednisolone and was tapered gradually. The patient was discharged on day 26 with prednisolone, 10 mg daily for 3 days. The patient was followed, and no evidence of development of chronic Q fever infective endocarditis was found by Q fever serological tests and echocardiography (Figure 2B).

Among the 11 tested cytokines, only IL-2, IL-6, IL-8, and IL-10 were detectable from disease onset to the 18-month follow-up. The levels of IL-2, IL-8, and IL-10 were elevated initially and decreased gradually, but the IL-6 level remained low (Figure 2C). The initial elevated PCT level also decreased with time (Figure 2D).

### The study and the patient

This study was approved by the Ethics Committee of the E-Da Hospital (EMRP-097-117). Signed informed consent was obtained from the patient before clinical observation and blood collection.

### Serologic assessments for specific antibodies against C. burnetii

Indirect immunofluorescence antibody assays (IFA) were performed for serologic assessment of specific antibodies against *C. burnetii *using commercially available kits (Focus Diagnostics Q Fever IFA IgG and IgM assay).

### Measurement of serum cytokines

The serum was separated from the blood within 2 hours after collection and was stored at -80°C until analysis. Eleven cytokines were tested simultaneously, including interferon-γ (INF-γ), interleukin-1β (IL-1β), IL-2, IL-4, IL-5, IL-6, IL-8, IL-10, IL-12 p70, tumor necrosis factor-α (TNF-α), and TNF-β using a multiplex cytokine kit (Human Th1/Th2 11plex FlowCytomix Multiplex [BMS810FF], Bender MedSystems™) and a BD FACSCalibur™ flow cytometer (Becton, Dickinson and Company) in accordance with the manufacturer's instructions. All assays were performed in duplicate.

### Measurement of serum procalcitonin *(PCT)*

Measurement of the serum PCT concentration was performed using VIDAS^® ^B·R·A·H·M·S PCT tests.

## Conclusions

Geographic differences are found between Q fever pneumonia and hepatitis [1], and the latter is predominant in Taiwan [5,6]. Cases of acute Q fever presenting with granulomatous hepatitis and persistent fever in spite of antimicrobial therapy that demanded combination therapy with steroids have been reported [2-4]. Among these reported cases, defervescence was usually achieved within a few days of initiation of steroid therapy (20-30 mg prednisolone/day) [2-4]. Immunologic and inflammatory process related fever due to development of autoantibodies were presumed responsible for the persistent fever [1-4]. In their review, Maurin and Raoult suggested a combination with a 6-day course of prednisolone when apyrexia was not obtained after 3 days of antibiotic therapy [1]. However, our patient did not defervesce until administration of intravenous methylprednisolone despite administration of a 6-day course of oral prednisolone. This indicates that the immunological response to *C. burnetii *infection in our patient might have been stronger, this requiring a higher dosage and a longer course of steroids to defervesce.

Previous studies investigating the cytokines involved in acute Q fever primarily focused on comparing patients with controls or those with different presentations, not on the response to treatment [7,8]. The circulating TNF and IL-6 levels were markedly increased in patients with acute Q fever compared with controls [7]. In the study of peripheral blood mononuclear cells (PBMCs) collected from acute Q fever patients, the production of TNF, IL-6, IL-12, and IL-10 was higher than in control subjects, and TNF and IL-10 levels were higher in patients with hepatitis than in those with isolated fever [8]. Induction of fever involves a multiple-pathway mechanism in which circulating cytokines, particularly proinflammatory cytokines including TNF, IL-1, IL-6, and INF, are the major mediators of fever [9]. However, only IL-2, IL-6, IL-8, and IL-10 were detectable in the serum of our patient (Figure 2C). Interleukin-2 is thought to induce fever through induction of IL-1 and TNF [9], and the persistent elevation of IL-2 might partially explain the difficulty in defervescence in our patient. Interleukin-6 has been proposed to be a mediator of fever downstream of IL-1 and TNF [9], but IL-6 persisted at a low level irrespective of fever in our patient. This result might reflect the undetectable serum levels of IL-1 and TNF. The fact that proinflammatory cytokines in our patient were undetectable might due to the inhibitory effect of an anti-inflammatory cytokine, IL-10, which was persistently elevated during the first month of illness and became undetectable 18 months later. Interleukin-8 and PCT are biomarkers used for predicting the severity of sepsis and mortality [10,11], but their kinetic change had not been investigated previously in acute Q fever patients. We found the initial level of PCT was increased (1.0 ng/ml) (< 0.05 ng/ml in health people), and it decreased gradually, as did the level of IL-8. Nevertheless, it must be remembered that the cytokine levels in the central nervous system rather than the circulating cytokine levels might be responsible for the persistent fever, which could explain why only a few cytokines were detected in the serum.

In conclusion, this case highlights the difficulty in treating acute Q fever when the patient responds poorly to standard antibiotic therapy. Cytokines might be associated with the persistence of fever in patients without defervescence despite antibiotic treatment. Procalcitonin might be used as an indicator for monitoring the response to treatment.

## Competing interests

All authors have seen and approved the manuscript and declare that they have no competing interest.

## Authors' contributions

CH drafted the manuscript. JN cared the patient, and provided information and discussed in writing manuscript. LL carried out the examinations of serum cytokines, procalcitonin, and *C. burnetii *antibodies. YH reviewed and revised the manuscript. HH designed and conducted the study. All authors read and approved the final manuscript.

## Pre-publication history

The pre-publication history for this paper can be accessed here:

http://www.biomedcentral.com/1471-2334/10/193/prepub
